# A study of the impact of staggered boards on corporate financialization: from the perspective of board governance

**DOI:** 10.3389/fpsyg.2024.1377948

**Published:** 2024-04-30

**Authors:** Chongyan Cao, Yutong Zhang

**Affiliations:** School of Management, University of Science and Technology of China, Hefei, Anhui, China

**Keywords:** staggered boards, corporate financialization, board governance, managerial ownership, audit supervision

## Abstract

**Introduction:**

The objective of this study is to assess the influence of staggered boards on corporate financialization and the role that incentive and supervision mechanisms play in this process.

**Methods:**

We employ a total of 20,647 panel data samples of Chinese A-share listed companies over the period 2011-2020 to empirically test the impact of staggered boards on corporate financialization in the Chinese context.

**Results:**

The results indicate that implementing staggered boards significantly increases levels of corporate financialization. On the one hand, the implementation of a staggered board structure can exacerbate the speculative mindset and profit-driven behavior among board members, leading management to prioritize financial investments for personal gain. On the other hand, a staggered board system may also amplify managerial laziness, potentially incentivizing them to rely heavily on financial investments in order to swiftly achieve performance targets with minimal effort. Furthermore, both managerial ownership and audit supervision are found to be critical factors in mitigating this positive impact and preventing excessive financial investment behavior.

**Discussion:**

This paper offers guidance on comprehending the applicability of staggered board provisions and mitigating financial risks in enterprises.

## 1 Introduction

In recent years, the decline in market demand and industrial overcapacity has resulted in an elevated risk of real investment, leading to a trend of “de-realization” in China’s real economy. Non-financial corporations are progressively reallocating their resources toward financial assets, thereby witnessing an intensified level of corporate financialization (Zhu et al., 2021; Fu et al., 2024). According to CSMAR data, the total amount of financial assets held by non-financial listed companies in China experienced a remarkable sixfold increase from 210.6 billion in 2011 to 1,519.8 billion in 2020.^1^ Furthermore, the average proportion of financial assets among non-financial listed companies witnessed a significant rise from approximately 1.82% in 2011 to 6.54% in 2020. Notably, certain real listed companies even observed their proportions of financial assets reaching as high as 77.84%. From a macro perspective, the real economy continues to serve as the foundation for a nation’s development; however, an influx of substantial capital into the virtual economic sector may hinder wealth creation and accumulation (Moosa, 2018; Ganic, 2023). From a micro perspective, excessive financialization has the potential to “squeeze out” investments in primary business operations, limit growth prospects for core businesses, and constrain long-term corporate development (Tori and Onaran, 2018; Leng et al., 2023). The issue of financialization within real firms has garnered significant attention from both governments and academics. Therefore, it is crucial to investigate the underlying motivations behind this phenomenon in order to effectively mitigate systemic financial risks.

Upon reviewing the literature, it is found that the financialization of real firms is essentially a financial investment behavior under the combined influence of the external economic environment (Perillo and Battiston, 2020; Zhao and Su, 2022; Yang et al., 2024) and the internal governance mechanism (Du et al., 2022; Feng et al., 2022). The board of directors, serving as the nucleus of internal governance and the supreme decision-making body within the organization, wields significant influence over the firm’s investment behavior (Dong et al., 2023). Given the intensifying competition in China’s control market for equity, it is increasingly prevalent for listed companies to incorporate provisions regarding staggered board clauses within their articles of association as a means to restrict the replacement of board members. Staggered board, also known as “classified board,” facilitates the gradual replacement of directors upon the expiration of their terms (Tanthanongsakkun et al., 2023). This is primarily observed in China through the inclusion of a provision in the articles of association that limits the proportion of directors to be replaced annually. Notably, this practice was initially introduced to ensure corporate governance stability and management continuity but has since been perceived as an anti-takeover mechanism, effectively mitigating the risk of board control transfers (Amihud et al., 2018a,b). Given the pivotal role played by staggered boards at the firm level, a plethora of studies have extensively examined their governance implications and increasingly focused on their impact on corporate investment behavior. For instance, Mbanyele (2021) conducted surveys among listed companies across six Asian nations and discovered that the implementation of staggered boards could hinder long-term R&D investments, consequently leading to diminished innovation efficiency. Conversely, Chen et al. (2022) ascertained that staggered boards can enhance the stability of corporate boards, positively influencing corporate product innovation and augmenting value-added long-term corporate investment.

Liu et al. (2023) point out that corporate governance can affect corporate financial investment by influencing managers’ behaviors. In fact, the staggered board system serves as a governance arrangement that objectively establishes a re-election guarantee and relative defense mechanism for directors of listed companies, thereby diminishing the monitoring of the external control market as well as the accountability mechanism of shareholders to board members (Mbanyele, 2021). It objectively mitigates the negative consequences of failed investment decisions on management careers and creates a managerial entrenchment (Faleye, 2007; Karakas and Mohseni, 2021). This entrenchment significantly influences decision-makers’ investment psychology and risk preferences, thereby affecting their choice between core business investments and financial investments, ultimately shaping the process of corporate financialization. However, there is a dearth of literature addressing the influence of the staggered boards on corporate financial investment behavior. In response to this gap, this paper empirically examines the impact of the staggered boards on corporate financialization in the context of the Chinese capital market from the perspective of board governance mechanisms using a sample of Chinese A-share listed companies from 2011 to 2020.

We selected Chinese listed firms as the sample for two significant reasons. Firstly, as a representative example of emerging market economies, China is currently undergoing a crucial stage of governance reform. Staggered board is regarded as an important innovative governance mechanism that Chinese enterprises can adopt. Through empirical findings in an institutional environment distinct from developed countries like the United States, this study can further increase the understanding of the staggered board system, especially increasing the understanding of the role played by the staggered board in emerging markets. Secondly, China possesses one of the world’s largest shadow banking industries (Ehlers et al., 2018). With China’s economy experiencing rapid growth, its financial system has expanded considerably (Yang et al., 2024). Investigating the relationship between staggered boards and corporate financialization can provide valuable insights into potential risks associated with this trend and hold relevance for other countries and regions with rapidly developing financial industries. This becomes particularly important in light of uncertainties in the international environment that may bring forth additional challenges to national governance innovation and vigilance against systemic financial risks (Fang and Ju, 2024).

Our study is an extension and complement to the research of Mbanyele (2021) and Chen et al. (2022) on the impact of staggered boards on firm investment, and we find that the establishment of staggered boards can significantly improve the level of corporate financialization, which is still stable after solving the endogeneity problem. This supports Mbanyele’s (2021) conclusion that staggered boards of directors negatively impact investment quality and efficiency.

We propose a mechanism that explains how staggered board systems impact corporate financialization. According to Liu et al. (2023), corporate governance can affect corporate financial investment by influencing managerial extravagant consumption and managerial effort. Our results support this view. The mechanism analysis shows that the establishment of staggered board provisions will aggravate the agency conflict between shareholders and managers, increase the explicit and implicit agency costs, and then lead to excessive investment in financial assets. On the one hand, staggered board will intensify the speculative psychology and profit motive of board members, causing management to use financial investment to seize private interests. On the other hand, staggered board system will also intensify the laziness psychology of management, potentially motivating management to use financial investment to quickly achieve the target performance without too much effort.

According to agency theory, incentive and supervision are important measures to optimize corporate governance (Dalton et al., 2007). We explore the efficacy of the governance role played by the equity incentive measure of management ownership and the audit supervision mechanism. It is found that both managerial ownership and audit supervision help to inhibit the promoting effect of staggered boards on corporate financialization, and improve the efficiency of corporate asset allocation. Furthermore, the advent of cutting-edge digital technologies is poised to revolutionize corporate governance through their seamless integration with enterprises (Li et al., 2024; Zhang and Wang, 2024). We find that digital transformation can significantly inhibit the promotion effect of staggered board on corporate financialization. Finally, the heterogeneity analysis finds that the nature of firm ownership and degrees of industry competition also affect the relationship between staggered boards and corporate financialization, which indicates the boundary conditions of staggered boards on corporate financialization.

The potential marginal contributions of this paper are as follows. Firstly, it expands the research scope on the factors influencing corporate financialization at the corporate governance level. Existing literature primarily focuses on institutional arrangements for shareholders (Shi et al., 2021; Jiang et al., 2022) and top management teams (Qi and Fang, 2023) when examining these factors, with less attention given to the impact of board governance mechanisms. This study addresses this gap by empirically investigating the relationship between staggered boards and corporate financialization, thereby enhancing research on factors influencing corporate financialization from a board governance perspective.

Secondly, this study aims to advance the research on staggered boards within the context of China’s capital market and emerging market (Wang et al., 2018; Chen et al., 2021). Considering that China’s capital and control markets are still in their nascent stage, there is a lag in implementing relevant corporate governance mechanisms and strengthening the legal system to meet actual developmental requirements (Ullah et al., 2023). Building upon prior research, this paper will also examine the suitability of staggered boards in China’s capital market as well as emerging market, and explore the circumstances under which they function effectively from a corporate finance perspective.

Finally, this study expands and examines the intermediary mechanism through which staggered boards influence corporate financialization, while also exploring the moderating impact of corporate incentive and supervision mechanisms. These findings offer valuable insights for harnessing the synergistic effects of staggered boards and corporate incentive and supervision mechanisms to mitigate any adverse consequences on firms.

The paper is structured as follows: Section 2 provides a theoretical analysis and formulates the research hypothesis. Section 3 describes the research design process in detail. Section 4 presents the empirical findings of the study. Section 5 explores the impact of digital transformation and conducts a heterogeneity analysis. Section 6 presents the conclusion and discussion.

## 2 Theoretical analysis and hypothesis development

In recent years, the staggered board system has gained increasing popularity among listed companies due to the increasingly active control rights market in China. The board of directors, as the cornerstone of corporate governance (Denis and McConnell, 2003), wields significant decision-making authority within the company (Asad et al., 2023; Dong et al., 2023). The implementation of a staggered board system objectively alters the tenure structure of directors, providing them with enhanced protection during their potential re-elections and thereby establishing substantial safeguards at the directorial level (Amihud et al., 2018a,b). Consequently, this influences investment motivations and decision-making preferences among directors, ultimately impacting their financial investment decisions.

Corporate decision-makers are incentivized to hold financial assets due to the high correlation between executives’ personal wealth and career success with the company’s business performance and stock price. The finance and real estate industries are perceived as having excessive profits, which can greatly attract enterprise managers with their short investment cycles and high return rates (Fu et al., 2024). This often leads to a reduction in investments in the entity’s main business and an allocation of more financial assets toward obtaining excess profits (Demir, 2009) while boosting short-term stock prices (Admati, 2017). The concern lies in the fact that while financial investments possess the potential to generate excessive returns, they often entail inherent risks and lack long-term sustainability (Xu and Xuan, 2021). Consequently, this can expose companies to financial distress and subsequent disciplinary threats such as management removal. In general, investment preferences of managers are often shaped by individual trade-offs between returns and risk. The staggered board system objectively undermines the governance role of the external control market and weakens the accountability mechanism of shareholders to directors (Bates et al., 2008; Bebchuk et al., 2009; Mbanyele, 2021), thereby fostering managerial entrenchment (Bebchuk and Cohen, 2005; Faleye, 2007; Karakas and Mohseni, 2021) and exacerbating the principal-agent problem between shareholders and managers. On the one hand, staggered boards allow entrenched managers to reap more private benefits at the expense of shareholders, and managers are more likely to allocate capital to financial assets that yield faster short-term returns in exchange for higher compensation. On the other hand, it should protect directors from the disciplinary threat of dismissal, weaken the negative impact of high risk of financial investment on the career of board members, further improve the tendency of financial investment of them, and thus improve the level of corporate financialization. Therefore, the following research hypothesis is proposed.

Hypothesis 1: Staggered boards can significantly promote corporate financialization.

Granting directors and other members of management a designated number of shares presents an efficacious remedy for the agency problem (Shan et al., 2019). Managerial shareholding confers upon management a residual claim, facilitating the alignment of residual control rights and claims within the enterprise, engendering interest convergence effects, fostering close integration of managerial interests with long-term enterprise development, and effectively mitigating managers’ opportunistic tendencies (Jensen and Meckling, 1976; Chen, 2005; Benkraiem et al., 2022). Managerial ownership can transform external supervision into a self-incentive and constraint, thereby serving as an essential alternative mechanism in cases where the existing supervision mechanism fails. The rationale behind this lies in the fact that equity incentives confer management with residual claim rights, while simultaneously exposing them to risks. Managers are required to bear the economic consequences of their decisions, and if they fail to effectively operate and consequently cause a decline in enterprise value, their personal income will also diminish (Fu et al., 2024). In enterprises with a staggered board system, although the agency problem is exacerbated due to the weakened supervisory mechanism, leading to financialization of the enterprise, the equity incentive mechanism compensates for this deficiency and mitigates agency conflicts as managerial shareholding increases (Lin, 2023). On the one hand, it expands the decision-making horizon of management and enhances the company’s long-term operational investment. On the other hand, it can curb managerial opportunism and mitigate intertemporal arbitrage in financial assets. This effectively constrains the positive association between staggered boards and corporate financialization. This leads to the following hypothesis.

Hypothesis 2: Managerial ownership can significantly impede the promotional impact of staggered boards on corporate financialization.

The financial statement audit of listed companies serves as a crucial external market supervision mechanism (Fargher et al., 2014). In cases where internal governance mechanisms fail to effectively constrain management behavior, external audit supervision can be employed as an alternative mechanism. During the process of financial statement auditing, auditors utilize their expertise and skills to assess the economic activities of enterprises in accordance with rigorous audit procedures. Statements audit can ensure the accuracy of a company’s financial information, effectively mitigate financial manipulation by management (Alzoubi, 2018), reduce the degree of information asymmetry between investors and management, and safeguard the rights and interests of investors (Dang et al., 2022). In general, reputable accounting firms are known to possess a more sophisticated supervision and audit system, providing a higher level of audit services and exerting a stronger external supervisory influence on management (Zahid et al., 2022). In order to uphold their own reputation, highly qualified auditors significantly reduce collusion with management, thereby enhancing the efficiency of supervision (Bacha et al., 2021). Moreover, superior quality audit services entail increased communication between auditors and the governance layer, leading to heightened attention and oversight from governance layer regarding management’s investment decisions. This contributes to improved effectiveness of internal control systems, further mitigating agency problems and constraining managerial behavior. Financial assets exhibit characteristics such as high returns and ease of transferability. Within the framework of risk-oriented auditing requirements, excessive allocation of financial assets by a company can impact an auditor’s assessment of the company’s sustainable operational capacity and subsequently influence their opinion in the audit report. Consequently, a higher level of audit service provided by an accounting firm leads to enhanced external supervision over management. This intensified external oversight prompts the management to reduce opportunistic behavior (Jiang et al., 2021) and prioritize the development of core business activities, thereby diminishing their utilization of financial assets for intertemporal arbitrage and mitigating the positive influence of staggered boards on corporate financialization. Therefore, the following research hypothesis is proposed.

Hypothesis 3: Audit supervision can significantly impede the promotional impact of staggered boards on corporate financialization.

## 3 Research design

### 3.1 Samples and data

We select Chinese A-share listed companies from 2011 to 2020 as the initial research sample, following these screening criteria (1) Exclude the financial and real estate listed companies, because we study the financialization of non-financial corporates. In recent years, the real estate sector has interacted frequently with the financial sector, and many studies discuss the real estate industry and the traditional financial industry collectively as the FIRE (finance, insurance, and real estate) (Krippner, 2005). Non-financial enterprises in this study refer to enterprises outside of the FIRE (2) Exclude the PT, ST or forcibly delisted companies, since our primary objective is to investigate the impact of staggered board systems on corporate financialization within normally operating listed firms. The operating conditions of specially treated listed companies are abnormal, resulting in abnormal holdings of financial assets. Excluding such samples ensures the effectiveness of econometric analysis by avoiding deviations in the analytical conclusions (3) Exclude samples with missing observed values. Ultimately, we observe a total of 3,141 listed companies with 20,647 samples. The sample screening process is shown in Table 1.

**Table 1:** Sample screening process.

Procedure	Method	Number of listed companies after screening	Number of samples after screening
1	All A-share listed companies in China	4,311	31,046
2	Excluding financial and real estate listed companies	4,068	28,992
3	Excluding companies that have been delisted and ST, PT	3,189	22,777
4	Excluding samples with missing data	3,141	20,647

Due to the absence of a dedicated database for staggered board provisions in China’s listed companies, we obtain the articles of association from Juchao Information Network for the years 2011 to 2020 and manually retrieve information on whether staggered board provisions are set. If a listed company’s articles of association contain provisions limiting director replacement proportions in a specific year, it is considered that staggered board provisions were established by that company during that year. The remaining data is sourced from the CSMAR database and Stata16.0 software is used for data analysis. Furthermore, to mitigate outliers’ influence on our results, all continuous variables underwent winsorization at the 1 and 99% levels prior to conducting descriptive statistics.

### 3.2 Model design and variable definition

In this paper, we construct model (1) to examine the impact of staggered boards on corporate financialization, while model (2) is developed to investigate the influence of managerial ownership and audit supervision on the association between staggered boards and corporate financialization.


(1) $$\begin{array}{l} Fin_{i,t}=\alpha_{0}+\alpha_{1}SBD_{i,t}+\sum \mathrm{Controls}\\ \;+\sum \mathrm{Industry}+\sum \mathrm{Year}+\mu_{i,t} \end{array}$$

(2) $$\begin{array}{l} Fin_{i,t}=\alpha_{0}+\alpha_{1}SBD_{i,t}+\alpha_{2}SBD_{i,t}\times M_{i,t}+\alpha_{3}M_{i,t}\\ \;+\alpha_{4}\sum \mathrm{Controls}+\sum \mathrm{Industry}+\sum \mathrm{Year}+\mu_{i,t} \end{array}$$

The dependent variable is corporate financialization (Fin). Orhangazi (2008) defines financialization as the increase of non-financial enterprises’ financial investment, the increase of the proportion of financial assets in total assets, and the increase of dependence on investment and financial activities rather than operating activities. Based on the definition of financial assets in Chinese Accounting Standard No. 22 (CAS22) and referring previous research (Demir, 2009; Tang and Zhang, 2019; Zou and Zhang, 2023; Zheng et al., 2024), this paper defines corporate financialization (Fin) as the ratio of financial assets to total assets. The higher the ratio, the higher the level of corporate financialization. Financial assets encompass trading financial assets, derivative financial assets, net loans and advances, available-for-sale securities, hold-to-maturity securities, and investment real estate. Monetary funds are excluded from our analysis as they primarily serve the daily operational needs of companies rather than generating profits (Jiang et al., 2022). Since modern real estate increasingly shows typical virtual characteristics and gradually separates from the real sector, this paper includes investment real estate into the category of financial assets (Zheng et al., 2024). We also adopt the absolute size of financial assets (LnFin) and a dummy variable (Fin_dummy) indicating whether the firm holds financial assets as the dependent variable to conduct robustness test. The absolute size of financial assets (LnFin) is measured by the natural logarithm of financial assets plus one.

The independent variable in this study pertains to the inclusion of staggered boards (SBD). Consistent with previous research (Wang et al., 2018; Chen et al., 2021; Tanthanongsakkun et al., 2023), a binary indicator is employed, whereby a value of 1 is assigned if the articles of association of a listed company incorporate the provision for staggered boards in a given year; otherwise, a value of 0 is assigned. The robustness test of this study also incorporates the staggered board limit ratio (SBLR) and the stringency score of staggered board limitation (SBscore) as alternative independent variables. If the company’s bylaws specify a maximum ratio for director replacements, the SBLR value represents that particular ratio. In cases where there are no restrictions on director replacements, the SBLR value is set to 1. Furthermore, the independent variables were reassigned based on the director replacement ratio limit in staggered board provision. The assignment value increases with a more stringent requirement for director replacement ratio. If the replacement ratio does not exceed 1/4, the SBscore value is assigned as 3. For replacement ratios greater than 1/4 but not exceeding 1/3, the SBscore value is assigned as 2. Similarly, if the replacement ratio exceeds 1/3 but does not exceed 2/3, the SBscore value is assigned as 1. In cases where there are no limits on change ratios, an SBscore value of 0 is assigned.

The regulatory variables encompass managerial ownership (Mshare) and audit supervision (Big4). According to Fu et al. (2024), managerial ownership (Mshare) is measured using the number of shares held by management as a proportion of the total number of shares in the firm. Audit supervision (Big4) is measured through a binary variable indicating whether the firm underwent an audit conducted by one of the Big 4 accounting firms at the end of the period. The reason for this is that the Big 4 firms have a greater incentive to provide high-quality audit services to maintain their reputation (Zahid et al., 2022). If a firm undergoes an audit conducted by one of the Big 4 accounting firms, it signifies that the firm has availed itself of top-tier audit service, thereby assigning a value of 1 to the audit supervision (Big4); otherwise, it is assigned a value of 0.

Referring to previous research (Jiang et al., 2022; Fu et al., 2024; Su et al., 2024), the control variables encompass firm size (Size), asset-liability ratio (Lev), operating cash flow (*CF*), profitability (ROA), fixed assets ratio (PPE), growth rate (Growth), enterprise value (TQ), listing age (Age), ownership concentration (First), institutional investor shareholding ratio (Ins), board size (Board), board independent (Dir), CEO duality (Dual). Size is the natural logarithm of the total assets. Lev is the ratio of total debts to total assets. *CF* is the ratio of operating cash flow to total assets. ROA is the ratio of net income to total assets. PPE is the proportion of fixed assets in the total assets. Growth is the growth rate of operating income. TQ is the market value to total assets. Age is the years of company experiencing since listing. First is the ratio of shareholdings for the biggest shareholder. Ins is the ratio of shareholdings for institutional investors. Board is the natural logarithm of the number of board members. Dir is the proportion of independent directors. Dual is a dummy variable. If the chairman and general manager hold the same position, the value is assigned as 1; otherwise, it is assigned as 0. We use a mixed OLS model for regression, which includes industry and year fixed effects. Refer to Table 2 for variable definitions.

**Table 2:** Variable definitions.

Type	Variable name	Symbol	Description
Dependent variable	Corporate financialization	*Fin*	(Financial assets)/(Total assets)
Independent variable	Staggered boards	*SBD*	If staggered board provisions are incorporated into the articles of association of a listed company in a given year, the assigned value is 1; otherwise, it is assigned a value of 0
Regulatory variables	Managerial ownership	Mshare	(Number of shares held by management)/ (Total enterprise shares)
	Audit supervision	Big4	If the firm undergoes an audit conducted by one of the Big 4 accounting firms, the assigned value is 1; otherwise, it is assigned a value of 0
Control variables	Firm size	*Size*	The natural logarithm of total assets
	Asset-liability ratio	*Lev*	Total liabilities/Total assets
	Operating cash flow	*CF*	(Net cash flows from operating activities)/ (Total assets)
	Profitability	*ROA*	(Net income)/ (Total assets)
	Fixed assets ratio	*PPE*	(Fixed assets)/ (Total assets)
	Growth	*Growth*	Growth rate of operating income
	Enterprise value	*TQ*	(Total market value)/ (Total assets)
	Listing age	*Age*	Years of company experience since listing
	Ownership concentration	*First*	(Number of shares held by the largest shareholder)/ (Total enterprise shares)*100%
	Institutional investor shareholding ratio	*Ins*	(Number of shares held by institutional investors)/ (Total enterprise shares)*100%
	Board size	*Board*	Natural logarithm of the number of board members
	Board independent	*Dir*	(Number of independent directors)/ (Total number of directors)
	CEO duality	*Dual*	If the chairman and general manager hold the same position, the value is assigned as 1; otherwise, it is assigned as 0

## 4 Empirical analysis

### 4.1 Descriptive statistics and correlation analysis

The descriptive statistical results of the variables are presented in Table 3. The financial asset allocation across different enterprises exhibits certain variations, with an average Fin value of 0.037, a standard deviation of 0.071, a minimum value of 0, and a maximum value of 0.398. Moreover, the analysis reveals that approximately only 4.6% of the listed companies have incorporated staggered board provisions into their articles of association (as indicated by the average SBD value being 0.046).

**Table 3:** Descriptive statistics.

Variables	N	Mean	Sd	Min	Mid	Max
Fin	20,647	0.037	0.071	0	0.007	0.398
SBD	20,647	0.046	0.208	0	0	1
Mshare	20,647	8.634	15.12	0	0.344	62.78
Big4	20,647	0.053	0.224	0	0	1
Size	20,647	22.09	1.232	20.00	21.91	26.04
Lev	20,647	0.392	0.195	0.048	0.383	0.832
*CF*	20,647	0.050	0.065	−0.136	0.049	0.228
ROA	20,647	0.044	0.052	−0.187	0.042	0.187
PPE	20,647	0.211	0.153	0.005	0.180	0.686
Growth	20,647	0.160	0.340	−0.496	0.107	2.011
TQ	20,647	1.988	1.156	0.870	1.610	7.471
Age	20,647	8.790	7.144	0	7	30
First	20,647	34.74	14.53	9.190	32.76	74.30
Ins	20,647	42.43	25.09	0.260	44.30	90.31
Board	20,647	2.126	0.199	1.099	2.197	2.890
Dir	20,647	0.375	0.055	0.167	0.333	0.800
Dual	20,647	0.301	0.459	0	0	1

The correlation analysis of the main variables indicates a significant and positive relationship between Fin and SBD, tentatively suggesting that the implementation of staggered boards intensifies corporate financialization. The correlation coefficients among these variables are all below 0.6, and further tests on Variance Inflation Factor (VIF) demonstrate VIF values below 3.000, effectively ruling out any substantial issues with multicollinearity. Detailed results can be found in Tables 4, 5.

**Table 4:** Correlation coefficients.

	Fin	SBD	Mshare	Big4	Size	Lev	*CF*	ROA	PPE	Growth	TQ	Age	First	Ins	Board	Dir	Dual
Fin	1																
SBD	0.062***	1															
Mshare	0.003	−0.072***	1														
Big4	−0.021***	−0.006	−0.097***	1													
Size	−0.021***	0.054***	−0.327***	0.357***	1												
Lev	−0.112***	0.055***	−0.272***	0.127***	0.551***	1											
*CF*	0.033***	−0.019***	−0.017**	0.080***	0.083***	−0.131***	1										
ROA	0.036***	−0.011	0.141***	0.027***	−0.065***	−0.378***	0.395***	1									
PPE	−0.183***	−0.024***	−0.187***	0.084***	0.189***	0.158***	0.238***	−0.089***	1								
Growth	−0.050***	−0.006	0.069***	−0.017**	0.022***	0.028***	−0.002	0.236***	−0.076***	1							
TQ	0.061***	0.003	0.046***	−0.085***	−0.339***	−0.291***	0.129***	0.220***	−0.137***	0.047***	1						
Age	0.113***	0.148***	−0.434***	0.092***	0.461***	0.369***	0.022***	−0.183***	0.164***	−0.094***	−0.069***	1					
First	−0.027***	−0.106***	−0.031***	0.126***	0.151***	0.030***	0.103***	0.126***	0.115***	−0.011	−0.074***	−0.048***	1				
Ins	−0.034***	0.003	−0.537***	0.233***	0.423***	0.211***	0.131***	0.080***	0.188***	0.009	−0.037***	0.255***	0.478***	1			
Board	−0.063***	0.024***	−0.176***	0.084***	0.270***	0.157***	0.032***	0.001	0.170***	−0.018***	−0.126***	0.171***	0.005	0.229***	1		
Dir	0.022***	−0.015**	0.111***	0.036***	−0.014**	−0.023***	−0.003	−0.013*	−0.060***	0.002	0.042***	−0.052***	0.044***	−0.086***	−0.555***	1	
Dual	0.030***	−0.029***	0.504***	−0.072***	−0.213***	−0.158***	−0.019***	0.053***	−0.133***	0.034***	0.070***	−0.258***	−0.030***	−0.200***	−0.190***	0.123***	1

Significance levels are denoted by ***, **, and * for 1, 5, and 10%, respectively.

**Table 5:** Variance inflation factor.

Variable	VIF	1/VIF
Size	2.150	0.464
Lev	1.800	0.557
Ins	1.700	0.589
Board	1.660	0.603
ROA	1.610	0.622
Dir	1.500	0.668
Age	1.480	0.677
First	1.410	0.709
*CF*	1.340	0.744
TQ	1.260	0.791
PPE	1.200	0.836
Dual	1.120	0.890
Growth	1.110	0.904
SBD	1.040	0.965
Mean VIF	1.460	

### 4.2 Regression analysis

Table 6 presents the results of mixed ordinary least squares (OLS) regressions on the impact of staggered boards on corporate financialization. Column (1) reports the regression result for the independent variable alone, while column (2) includes additional controls for industry and year fixed effects. In column (3), control variables are added without including industry and year fixed effects, and in column (4), both control variables and industry/year fixed effects are included. The coefficients of SBD in columns (1)–(4) are 0.021, 0.017, 0.013, and 0.013 respectively, all statistically significant at the 1% level. This confirms Hypothesis 1 and provides evidence that staggered boards lead to managerial entrenchment effect and promote corporate financialization.

**Table 6:** Regression results.

Variables	(1)	(2)	(3)	(4)	(5)	(6)	(7)	(8)
	Fin	Fin	Fin	Fin	Fin_l	Fin_s	Fin	Fin
SBD	0.021***	0.017***	0.013***	0.013***	0.002	0.011***	0.015***	0.014***
	(7.68)	(6.16)	(5.20)	(5.06)	(1.59)	(5.29)	(5.48)	(5.18)
SBD*Mshare							−0.001**	
							(−2.21)	
Mshare							−0.000**	
							(−2.31)	
SBD*Big4								−0.017*
								(−1.88)
Big4								−0.002
								(−1.18)
Size			0.002***	0.000	−0.002***	0.002***	0.001	0.001
			(3.63)	(0.90)	(−8.63)	(5.02)	(1.04)	(1.22)
Lev			−0.056***	−0.059***	−0.002	−0.055***	−0.059***	−0.059***
			(−16.49)	(−16.90)	(−1.51)	(−18.43)	(−17.02)	(−16.92)
*CF*			0.073***	0.023***	0.009***	0.011	0.023***	0.023***
			(8.45)	(2.73)	(2.59)	(1.59)	(2.78)	(2.76)
ROA			−0.028**	0.007	−0.020***	0.026**	0.010	0.007
			(−2.22)	(0.57)	(−3.80)	(2.43)	(0.78)	(0.56)
PPE			−0.095***	−0.073***	−0.014***	−0.054***	−0.074***	−0.073***
			(−29.81)	(−19.57)	(−8.51)	(−17.40)	(−19.70)	(−19.57)
Growth			−0.008***	−0.005***	−0.001	−0.004***	−0.005***	−0.005***
			(−5.77)	(−3.35)	(−1.46)	(−3.25)	(−3.21)	(−3.41)
TQ			0.000	0.000	−0.000**	0.001*	0.000	0.000
			(0.46)	(0.46)	(−2.55)	(1.71)	(0.41)	(0.52)
Age			0.002***	0.001***	0.001***	0.001***	0.001***	0.001***
			(21.19)	(15.73)	(21.16)	(7.56)	(14.91)	(15.62)
First			0.000**	0.000***	0.000***	0.000**	0.000***	0.000***
			(2.53)	(3.71)	(3.27)	(2.19)	(4.35)	(3.75)
Ins			−0.000***	−0.000***	−0.000***	−0.000**	−0.000***	−0.000***
			(−3.22)	(−3.28)	(−3.39)	(−2.39)	(−4.18)	(−3.19)
Board			−0.017***	−0.010***	−0.006***	−0.005*	−0.010***	−0.010***
			(−5.54)	(−3.36)	(−4.76)	(−1.91)	(−3.13)	(−3.35)
Dir			−0.021**	−0.014	−0.007**	−0.005	−0.013	−0.014
			(−2.10)	(−1.45)	(−1.97)	(−0.66)	(−1.34)	(−1.42)
Dual			0.004***	0.002*	0.000	0.001	0.004***	0.002*
			(3.83)	(1.78)	(0.70)	(1.38)	(2.69)	(1.81)
Constant	0.036***	0.012*	0.061***	0.052***	0.057***	−0.007	0.050***	0.047***
	(70.72)	(1.66)	(4.81)	(3.37)	(10.82)	(−0.54)	(3.23)	(2.95)
Industry	NO	YES	NO	YES	YES	YES	YES	YES
Year	NO	YES	NO	YES	YES	YES	YES	YES
*N*	20,647	20,647	20,647	20,647	20,647	20,647	20,647	20,647
Adj-*R*^2^	0.004	0.115	0.085	0.160	0.163	0.125	0.161	0.160

Significance levels are denoted by ***, **, and * for 1, 5, and 10% respectively; *t*-values are presented in parentheses.

Additionally, we classify financial assets into short-term and long-term categories based on their liquidity, and further examine the impact of staggered boards on the allocation of these different types of financial assets. Financial assets with high liquidity, such as trading financial assets, net loans and advances, and net financial assets available for sale, are categorized as short-term financial assets (Fin_s), while those with longer maturities like derivative financial assets, net hold-to-maturity investments, and net investment in real estate are classified as long-term financial assets (Fin_l). The regression results presented in columns (5) and (6) of Table 6 demonstrate a more significant effect of staggered boards on short-term financial investments. This suggests that protected directors tend to prefer investing in shorter maturity financial assets due to their higher liquidity levels, lower risk profiles, shorter return cycles, and greater convenience for manipulation or earnings management.

The test results of the moderating effect of managerial ownership (Mshare) and audit supervision (Big4) are presented in Columns (7) and (8) of Table 6. In Column (7), the coefficient of the interaction term (SBD* Mshare) is −0.001, which is statistically significant at a 5% significance level. This indicates that as management shareholding increases, the positive impact of staggered boards on corporate financialization will be suppressed, thus confirming Hypothesis 2. In Column (8), the coefficient of the interaction term (SBD* Big4) is −0.017, which is statistically significant at a 10% significance level. This suggests that high-level audit supervision can effectively inhibit the positive impact of staggered boards on corporate financialization, thus confirming Hypothesis 3.

### 4.3 Mechanism analysis

According to agency theory, in the contemporary corporate structure characterized by a separation of control and ownership, both shareholders and managers strive to maximize their individual utility. In the absence of an effective monitoring mechanism, agency problems can easily arise. The implementation of staggered boards in firms objectively diminishes shareholders’ ability to monitor directors, increasing the difficulty and cost associated with replacing directors (Mbanyele, 2021). Consequently, even if directors make decisions that deviate from the maximization of shareholder value, shareholders are unable to promptly remove them, thereby affording directors protection against accountability and exacerbating the agency conflict between shareholders and management (Field and Lowry, 2022). On one hand, staggered boards enable entrenched and ineffective managers to enhance their personal benefits at the expense of shareholders. Managers driven by profit-seeking motives may indulge in excessive on-the-job consumption, engage in non-essential expenditures, and exploit private benefits (Karakas and Mohseni, 2021), thereby amplifying the explicit agency costs of enterprises. Financial assets characterized by robust liquidity, high returns, and streamlined earnings management are increasingly prone to serve as a significant mechanism for rent-seeking by management to secure private control benefits. On the other hand, the heightened agency conflict can also manifest as a significant decrease in managerial effort and inclination toward maintaining a “quiet life” (Bertrand and Mullainathan, 2003; Chintrakarn et al., 2013), leading to inefficient investments and subsequently increased implicit agency costs. Given the potential for significant returns within a short timeframe, financial investments provide managers with an accelerated pathway to attaining their desired performance levels with minimal effort. Consequently, heightened agency conflicts between shareholders and directors may exacerbate decision makers’ financial investment preferences and amplify the phenomenon of corporate financialization.

Based on the aforementioned analysis, this study posits that staggered boards exacerbate the agency conflict between shareholders and managers, thereby augmenting the level of corporate financialization. Models (3) and (4) are employed to examine the role of agency conflict in elucidating the impact of staggered boards on corporate financialization.


(3) $$\begin{array}{l} \mathrm{Agencycos}t_{i,t}=\alpha_{0}+\alpha_{1}SBD_{i,t}+\sum \mathrm{Con}t\mathrm{rols}\\ \;+\sum \mathrm{Industry}+\sum \mathrm{Year}+\mu_{i,t} \end{array}$$

(4) $$\begin{array}{l} {\mathrm{Fin}}_{i,t}=\alpha_{0}+\alpha_{1}{SBD}_{i,t}+\alpha_{2}{\mathrm{Agencycost}}_{i,t}\\ \;+\alpha_{3}\sum \mathrm{Controls}+\sum \mathrm{Industry}+\sum \mathrm{Year}+\mu_{i,t} \end{array}$$

Referring to the research of Ang et al. (2000) and Purkayastha et al. (2022), we utilize the ratio of selling expenses and management expenses to operating revenue as a measure of explicit agency cost (Sale). Additionally, we use total asset turnover (TAT) as an indicator for implicit agency cost. It is important to note that a lower total asset turnover signifies a higher level of implicit agency cost. The results of the mechanism analysis are presented in Table 7. Columns (2) and (3) indicate that staggered boards significantly increase the sales management expense rate of enterprises, suggesting that after introducing staggered board provisions, on-the-job consumption and unnecessary expenditures by management greatly increase, resulting in explicit agency costs for enterprises and a greater tendency for management to use financial assets to pursue private interests. Columns (4) and (5) demonstrate that establishing staggered boards significantly reduces managers’ effort, thereby increasing implicit agency costs for firms. As a result, there is a marked preference for investing in financial assets characterized by high returns with minimal effort, further escalating corporate financialization.

**Table 7:** Mechanism test results.

Variables	(1)	(2)	(3)	(4)	(5)
	Fin	Sale	Fin	TAT	Fin
SBD	0.013*** (5.06)	0.009** (2.33)	0.013*** (5.02)	−0.052*** (−3.78)	0.012*** (4.66)
Sale			0.011* (1.81)		
TAT					−0.020*** (−13.85)
Size	0.000	−0.004***	0.001	−0.039***	−0.000
	(0.90)	(−4.98)	(0.98)	(−12.02)	(−0.53)
Lev	−0.059***	−0.140***	−0.057***	0.712***	−0.044***
	(−16.90)	(−26.80)	(−16.09)	(36.52)	(−12.62)
*CF*	0.023***	0.082***	0.022***	0.586***	0.035***
	(2.73)	(6.33)	(2.62)	(10.47)	(4.14)
ROA	0.007	−0.421***	0.012	1.763***	0.043***
	(0.57)	(−19.91)	(0.92)	(25.45)	(3.27)
PPE	−0.073***	−0.074***	−0.073***	−0.229***	−0.078***
	(−19.57)	(−12.73)	(−19.35)	(−10.15)	(−20.57)
Growth	−0.005***	−0.020***	−0.005***	0.099***	−0.003*
	(−3.35)	(−8.13)	(−3.20)	(10.25)	(−1.95)
TQ	0.000	0.013***	0.000	−0.013***	0.000
	(0.46)	(14.51)	(0.22)	(−4.77)	(0.02)
Age	0.001***	−0.000	0.001***	0.002***	0.001***
	(15.73)	(−0.96)	(15.75)	(2.99)	(16.18)
First	0.000***	−0.000*	0.000***	0.001***	0.000***
	(3.71)	(−1.88)	(3.74)	(6.19)	(4.35)
Ins	−0.000***	−0.000***	−0.000***	0.001***	−0.000***
	(−3.28)	(−6.64)	(−3.17)	(7.13)	(−2.62)
Board	−0.010***	0.012***	−0.011***	0.008	−0.010***
	(−3.36)	(2.64)	(−3.41)	(0.51)	(−3.32)
Dir	−0.014	0.056***	−0.015	−0.005	−0.014
	(−1.45)	(3.85)	(−1.51)	(−0.10)	(−1.46)
Dual	0.002*	0.006***	0.002*	−0.021***	0.002
	(1.78)	(4.01)	(1.72)	(−3.85)	(1.41)
Constant	0.052***	0.296***	0.049***	0.984***	0.072***
	(3.37)	(13.98)	(3.14)	(12.52)	(4.64)
Industry	YES	YES	YES	YES	YES
Year	YES	YES	YES	YES	YES
*N*	20,647	20,647	20,647	20,647	20,647
Adj-*R*^2^	0.160	0.459	0.160	0.371	0.170

Significance levels are denoted by ***, **, and * for 1, 5, and 10% respectively; *t*-values are presented in parentheses.

### 4.4 Endogeneity test

This study inevitably faces endogeneity issues, including omitted variables, reverse causality, and sample self-selection. We try to address these concerns by using individual fixed effect model, lagged variable method, propensity score matching (PSM) analysis, Heckman two-stage model, and instrumental variable (IV) approach.

Using Individual Fixed Effect Model. Our model may not be able to control all the factors that affect corporate financialization, which leads to the endogeneity problem of omitted variables. To address it, we employ an individual fixed effect model, which is regarded as an effective solution (Amihud et al., 2018a,b; Tanthanongsakkun et al., 2023), to further examine the hypothesis (refer to Table 8 for results). The findings indicate that the coefficient of SBD remains significantly positive, aligning with the aforementioned empirical outcomes.

**Table 8:** Regression results of fixed effect model and model of lagged independent and control variables.

Variables	Fixed effect model				Lagging one period				Lagging two period			
	(1)	(2)	(3)	(4)	(5)	(6)	(7)	(8)	(9)	(10)	(11)	(12)
	Fin	Fin	Fin	Fin	Fin	Fin	Fin	Fin	Fin	Fin	Fin	Fin
SBD	0.014**	0.017***	0.017***	0.017***	0.023***	0.017***	0.015***	0.013***	0.023***	0.017***	0.015***	0.012***
	(2.29)	(2.77)	(2.79)	(2.88)	(7.64)	(5.81)	(5.16)	(4.55)	(7.16)	(5.20)	(4.95)	(4.09)
Size			−0.003*	−0.003			0.001	0.000			0.001	0.000
			(−1.67)	(−1.22)			(1.58)	(0.34)			(0.80)	(0.25)
Lev			−0.011*	−0.014**			−0.051***	−0.055***			−0.052***	−0.055***
			(−1.76)	(−2.12)			(−13.50)	(−14.20)			(−12.00)	(−12.41)
*CF*			−0.006	−0.011			0.088***	0.049***			0.092***	0.061***
			(−0.76)	(−1.43)			(9.14)	(5.16)			(8.15)	(5.56)
ROA			−0.028**	−0.028**			−0.023	0.005			−0.040**	−0.024
			(−2.14)	(−2.09)			(−1.53)	(0.34)			(−2.05)	(−1.20)
PPE			−0.042***	−0.040***			−0.088***	−0.068***			−0.089***	−0.071***
			(−5.31)	(−5.06)			(−24.69)	(−15.86)			(−21.92)	(−14.84)
Growth			0.000	0.001			−0.007***	−0.006***			−0.007***	−0.008***
			(0.21)	(0.87)			(−4.29)	(−3.59)			(−3.88)	(−4.41)
TQ			0.005***	0.005***			−0.001	−0.000			−0.001*	−0.000
			(7.62)	(6.58)			(−1.40)	(−0.59)			(−1.84)	(−0.61)
Age			0.005***	0.005***			0.002***	0.002***			0.002***	0.001***
			(13.79)	(12.92)			(20.99)	(16.02)			(17.65)	(13.11)
First			0.000**	0.000**			0.000***	0.000***			0.000***	0.000***
			(2.20)	(2.20)			(3.32)	(4.15)			(2.89)	(3.38)
Ins			−0.000***	−0.000***			−0.000***	−0.000***			−0.000***	−0.000***
			(−3.86)	(−4.03)			(−4.89)	(−4.98)			(−4.70)	(−4.94)
Board			−0.005	−0.006			−0.017***	−0.011***			−0.014***	−0.010***
			(−0.81)	(−0.95)			(−4.99)	(−3.38)			(−3.85)	(−2.61)
Dir			−0.022	−0.022			−0.016	−0.014			−0.006	−0.007
			(−1.38)	(−1.42)			(−1.46)	(−1.27)			(−0.50)	(−0.58)
Dual			0.001	0.001			0.004***	0.003**			0.003**	0.002
			(0.38)	(0.43)			(3.60)	(2.10)			(2.50)	(1.38)
Constant	0.036***	0.016***	0.091*	0.080*	0.036***	0.016*	0.077***	0.062***	0.038***	0.019*	0.083***	0.063***
	(129.38)	(15.10)	(1.93)	(1.69)	(66.55)	(1.82)	(5.53)	(3.62)	(63.59)	(1.85)	(5.43)	(3.29)
Firm	YES	YES	YES	YES	NO	NO	NO	NO	NO	NO	NO	NO
Industry	NO	NO	NO	NO	NO	YES	NO	YES	NO	YES	NO	YES
Year	NO	YES	NO	YES	NO	YES	NO	YES	NO	YES	NO	YES
*N*	20,647	20,647	20,647	20,647	16,867	16,867	16,867	16,867	14,137	14,137	14,137	14,137
Adj-*R*^2^	0.001	0.073	0.078	0.083	0.005	0.107	0.085	0.153	0.005	0.098	0.077	0.142

Significance levels are denoted by ***, **, and * for 1, 5, and 10% respectively; *t*-values are presented in parentheses.

Lagged variable method. In this study, the model cannot exclude the problem of reverse causality because the dependent variable and independent variable are in the same period. Therefore, we employ a lagged independent variable and control variables by one or two periods to examine the robustness of our previous findings (refer to Table 8 for details). The results consistently demonstrate a significantly positive coefficient of SBD at the 1% level in both regressions, excluding the reverse causality problem and confirming the robustness of our empirical outcomes.

Propensity score matching. Given that the proportion of companies with staggered boards in our sample is only 4.6%, significantly smaller than the proportion of companies without staggered boards, we employ Propensity Score Matching (PSM) analysis to address potential noise and endogeneity concerns (Lennox et al., 2012; Tanthanongsakkun et al., 2023). Initially, we select control variables from the benchmark model as key factors for estimating the likelihood of adopting staggered boards. Subsequently, a neighbor matching method is employed to match every treatment group sample with 2 samples without staggered boards to form the control group. The matching results obtained through PSM are presented in Table 9, where all matched pairs exhibit an absolute standard deviation below 10% and none of them yield significant t-test results, indicating satisfactory matching outcomes. Finally, the matching samples are used to regress Model (1). Moving forward to Table 10, we present the regression results after propensity score matching, where it is noteworthy that the coefficients associated with staggered board (SBD) remain consistently positive and highly significant.

**Table 9:** Results of the equilibrium test for PSM.

Variables		Mean		% Bias	% Reduct |bias|	*t*	*p* > *t*
		Treat	Control				
Size	Unmatched	22.401	22.08	27.1	97.8	7.83	0.000
	Matched	22.401	22.39	0.6		0.13	0.898
Lev	Unmatched	0.441	0.390	27.1	98.7	7.96	0.000
	Matched	0.441	0.442	−0.4		−0.08	0.937
*CF*	Unmatched	0.044	0.050	−9.0	54.4	−2.72	0.007
	Matched	0.044	0.042	4.1		0.88	0.379
ROA	Unmatched	0.042	0.044	−5.4	88.4	−1.60	0.110
	Matched	0.042	0.042	−0.6		−0.14	0.890
PPE	Unmatched	0.195	0.212	−11.3	72.6	−3.41	0.001
	Matched	0.195	0.190	3.1		0.69	0.493
Growth	Unmatched	0.152	0.161	−2.7	31.6	−0.80	0.424
	Matched	0.152	0.158	−1.8		−0.38	0.703
TQ	Unmatched	2.01	1.985	1.6	−7.7	0.50	0.620
	Matched	2.01	1.986	1.7		0.35	0.724
Age	Unmatched	13.618	8.56	71.0	99.9	21.44	0.000
	Matched	13.618	13.63	−0.1		−0.02	0.983
First	Unmatched	27.667	35.075	−53.6	97.9	−15.36	0.000
	Matched	27.667	27.821	−1.1		−0.26	0.795
Ins	Unmatched	42.76	42.41	1.5	7.3	0.42	0.675
	Matched	42.76	42.44	1.4		0.34	0.732
Board	Unmatched	2.148	2.125	11.4	58.4	3.44	0.001
	Matched	2.148	2.157	−4.8		−1.03	0.305
Dir	Unmatched	0.371	0.375	−6.9	66.2	−2.11	0.035
	Matched	0.371	0.370	2.3		0.52	0.606
Dual	Unmatched	0.239	0.304	−14.5	86.0	−4.21	0.000
	Matched	0.239	0.248	−2.0		−0.46	0.648

**Table 10:** Regression results of PSM and Heckman two-stage model.

Variables	PSM	Heckman two-stage model	
		First Stage	Second Stage
	Fin	SBD	Fin
SBD	0.012***		0.012***
	(3.95)		(4.60)
imr			0.097***
			(3.10)
Size	−0.001	0.084***	0.008***
	(−0.41)	(3.98)	(3.20)
Lev	−0.060***	−0.064	−0.064***
	(−5.42)	(−0.55)	(−16.03)
*CF*	0.050**	−0.209	0.005
	(2.00)	(−0.68)	(0.49)
ROA	0.029	0.831*	0.076***
	(0.72)	(1.89)	(2.95)
PPE	−0.099***	−0.294**	−0.099***
	(−8.64)	(−2.01)	(−10.68)
Growth	−0.007*	−0.033	−0.007***
	(−1.94)	(−0.65)	(−4.40)
TQ	0.002	0.024	0.002***
	(0.93)	(1.37)	(2.67)
Age	0.002***	0.044***	0.005***
	(8.45)	(15.51)	(4.24)
First	−0.000	−0.021***	−0.002***
	(−0.97)	(−13.02)	(−2.83)
Ins	−0.000***	0.002**	0.000*
	(−2.58)	(2.39)	(1.70)
Board	−0.006	−0.207*	−0.028***
	(−0.71)	(−1.93)	(−4.34)
Dir	−0.011	−0.481	−0.055***
	(−0.36)	(−1.32)	(−3.37)
Dual	0.003	0.026	0.004***
	(0.97)	(0.66)	(3.17)
Constant	0.088**	−1.801***	−0.164**
	(2.08)	(−3.29)	(−2.27)
Industry	YES	YES	YES
Year	YES	YES	YES
*N*	2,589	20,647	20,647
Adj-*R*^2^	0.193		0.161

Significance levels are denoted by ***, **, and * for 1, 5, and 10% respectively; *t*-values are presented in parentheses.

Heckman two-stage model. The potential issue with the empirical findings lies in the possibility of self-selection, as managers who anticipate utilizing financial asset allocation for personal gains or employing financial investments to mask their lackluster performance may opt to incorporate staggered board provisions into their company’s articles of association as a safeguard against dismissal. Consequently, the observed positive correlation between staggered boards and corporate financialization might be attributed to the inclination of managers with a higher degree of financial asset allocation to adopt staggered boards within their organizations for protective purposes. In order to address the issue of sample self-selection bias in empirical findings, we employ the Heckman two-stage method. In the first step, a probit regression model is constructed using a dummy variable SBD to estimate the probability of firms setting the staggered board system. Subsequently, based on the regression results, we calculate the Inverse Mills Ratio (imr). In the second step, we incorporate imr as a control variable into the benchmark model for regression analysis (refer to Table 10 for details). The findings demonstrate that even after accounting for sample selection bias, the coefficient of SBD remains significantly positive at a 1% level of significance, thus confirming robustness in our empirical conclusions.

IV-2SLS. We adopt the instrumental variable approach to further address the endogeneity problem in the study. Drawing on the practice of Tanthanongsakkun et al. (2023) and Chatjuthamard et al. (2024), we adopt the instrumental variable based on geographical location. we utilize the average staggered board limit ratio (SBLR) of other companies in the same city (CITY_SBLR) and the average stringency score of staggered board limitation (SBscore) of other companies in the same city (CITY_SBscore) as instrumental variables. Companies that are geographically adjacent may have similar attributes in terms of investor clientele, local competition, and social connections, and therefore may adopt similar governance arrangements. Furthermore, as highlighted by Tanthanongsakkun et al. (2023), the selection of a company’s headquarters is typically determined in its early stages, often unrelated to the current characteristics of the company, thus representing an exogenous variable. Table 11 reports the regression results. The regression results of the first stage show that the coefficient of CITY_SBLR is significantly negative, while the coefficient of CITY_SBscore is significantly positive, indicating that companies in the same geographical location have a consistent tendency to set staggered board system. The regression results of the second stage show that that staggered board improves the level of corporate financialization, which is consistent with the empirical results.

**Table 11:** Regression result of IV.

Variables	CITY_SBLR		CITY_SBscore	
	First Stage	Second Stage	First Stage	Second Stage
	SBD	Fin	SBD	Fin
CITY_SBLR	−0.173***			
	(−23.20)			
CITY_SBscore			0.037***	
			(3.57)	
SBD		0.025**		0.113*
		(2.22)		(1.65)
Size	0.003	0.000	0.005**	−0.000
	(1.51)	(0.68)	(2.56)	(−0.03)
Lev	0.015	−0.061***	0.006	−0.061***
	(1.47)	(−16.81)	(0.58)	(−16.21)
*CF*	−0.026	0.024***	−0.027	0.026***
	(−0.97)	(2.78)	(−0.99)	(2.86)
ROA	0.133***	0.007	0.118***	−0.003
	(3.83)	(0.53)	(3.35)	(−0.21)
PPE	0.007	−0.074***	−0.015	−0.073***
	(0.49)	(−19.07)	(−1.07)	(−16.93)
Growth	−0.007*	−0.005***	−0.007	−0.004***
	(−1.66)	(−3.29)	(−1.45)	(−2.69)
TQ	0.001	0.000	0.002	0.000
	(0.51)	(0.65)	(1.22)	(0.33)
Age	0.004***	0.001***	0.004***	0.001***
	(14.02)	(14.06)	(14.13)	(3.57)
First	−0.001***	0.000***	−0.001***	0.000***
	(−12.60)	(4.08)	(−12.26)	(2.95)
Ins	0.000*	−0.000***	0.000	−0.000***
	(1.82)	(−2.97)	(1.30)	(−3.10)
Board	−0.004	−0.011***	−0.012	−0.010***
	(−0.40)	(−3.51)	(−1.19)	(−2.97)
Dir	−0.045	−0.014	−0.056	−0.010
	(−1.30)	(−1.40)	(−1.58)	(−0.85)
Dual	0.002	0.002	0.005	0.001
	(0.67)	(1.60)	(1.62)	(1.11)
Constant	0.349***	0.055***	0.130**	0.043**
	(5.40)	(3.45)	(1.97)	(2.31)
Industry	YES	YES	YES	YES
Year	YES	YES	YES	YES
*N*	19,630	19,630	19,630	19,630
Underidentification test: Kleibergen-Paap rk LM statistic	515.44		12.65	
Weak identification Test: Cragg-Donald Wald *F*-statistic	700.216		21.52	

Significance levels are denoted by ***, **, and * for 1, 5, and 10% respectively; *t*-values are presented in parentheses.

### 4.5 Robustness test

Replacing the dependent variable. In order to enhance the reliability of our research findings, we introduce additional variables to measure corporate financialization. Specifically, we include a dummy variable (Fin_dummy) and an absolute scale indicator of financial assets (LnFin). The corresponding results are presented in Table 12. Columns (1), (2), and (4) demonstrate significantly positive coefficients for SBD, while column (3) exhibits a positive but statistically insignificant coefficient. This implies that firms with staggered boards are more likely to possess financial assets. Moreover, when examining LnFin across columns (5)–(8), all coefficients for SBD remain significantly positive, thus confirming the robustness of our findings.

**Table 12:** Regression results of replacing the dependent variable.

Variables	(1)	(2)	(3)	(4)	(5)	(6)	(7)	(8)
	Fin_dummy	Fin_dummy	Fin_dummy	Fin_dummy	LnFin	LnFin	LnFin	LnFin
SBD	0.112***	0.104***	0.015	0.026**	2.709***	2.500***	0.662***	0.808***
	(9.04)	(8.52)	(1.30)	(2.19)	(11.33)	(10.88)	(3.03)	(3.77)
Size			0.100***	0.085***			2.496***	2.242***
			(32.17)	(25.27)			(44.61)	(37.28)
Lev			−0.038*	−0.012			−2.000***	−1.646***
			(−1.95)	(−0.59)			(−5.78)	(−4.61)
*CF*			0.383***	0.238***			7.888***	4.631***
			(7.27)	(4.52)			(8.47)	(5.01)
ROA			−0.372***	−0.159**			−7.264***	−2.992**
			(−5.33)	(−2.26)			(−5.81)	(−2.38)
PPE			−0.299***	−0.187***			−7.479***	−4.950***
			(−14.51)	(−7.33)			(−20.34)	(−11.04)
Growth			−0.013	−0.013			−0.393**	−0.364**
			(−1.53)	(−1.54)			(−2.57)	(−2.44)
TQ			0.027***	0.012***			0.465***	0.191***
			(9.68)	(3.84)			(9.36)	(3.47)
Age			0.011***	0.009***			0.232***	0.191***
			(25.45)	(19.97)			(28.82)	(22.36)
First			−0.001***	−0.000*			−0.014***	−0.005
			(−3.50)	(−1.78)			(−3.17)	(−1.20)
Ins			−0.001***	−0.001***			−0.018***	−0.013***
			(−5.64)	(−3.73)			(−6.48)	(−4.79)
Board			−0.022	0.051***			−0.722**	0.624**
			(−1.24)	(2.88)			(−2.29)	(1.98)
Dir			−0.057	0.063			−0.771	1.211
			(−0.94)	(1.05)			(−0.71)	(1.14)
Dual			0.013*	0.003			0.325***	0.116
			(1.94)	(0.45)			(2.74)	(1.00)
Constant	0.729***	0.528***	−1.421***	−1.440***	13.059***	8.979***	−39.648***	−40.214***
	(230.07)	(11.52)	(−19.09)	(−16.10)	(223.59)	(10.90)	(−29.51)	(−25.39)
Industry	NO	YES	NO	YES	NO	YES	NO	YES
Year	NO	YES	NO	YES	NO	YES	NO	YES
*N*	20,647	20,647	20,647	20,647	20,645	20,645	20,645	20,645
Adj-*R*^2^	0.003	0.107	0.130	0.185	0.005	0.138	0.199	0.261

Significance levels are denoted by ***, **, and * for 1, 5, and 10% respectively; *t*-values are presented in parentheses.

Replacing independent variables. The Staggered Board Limit Ratio (SBLR) and the stringency score of staggered board limitation (SBscore) are employed as the independent variable for conducting a robustness test. See Table 13 for details. The coefficients of SBLR are all significantly negative at the level of 1%, indicating that a smaller proportion of restricted annual replacements corresponds to stronger enforcement of staggered boards and higher levels of corporate financialization. The coefficients of SBscore exhibit a significant positive association at the 1% level, indicating that a more stringent requirement for director replacement ratio leads to enhanced director protection and an increased degree of corporate financialization.

**Table 13:** Regression results of replacing the independent variable.

Variables	(1)	(2)	(3)	(4)	(5)	(6)	(7)	(8)
	Fin	Fin	Fin	Fin	Fin	Fin	Fin	Fin
SBLR	−0.031***	−0.024***	−0.020***	−0.019***				
	(−7.64)	(−6.19)	(−5.21)	(−5.08)				
SBscore					0.009***	0.007***	0.006***	0.005***
					(7.39)	(5.93)	(5.04)	(4.84)
Size			0.002***	0.000			0.002***	0.001
			(3.63)	(0.90)			(3.63)	(0.91)
Lev			−0.056***	−0.059***			−0.056***	−0.059***
			(−16.48)	(−16.89)			(−16.46)	(−16.88)
*CF*			0.073***	0.023***			0.073***	0.023***
			(8.45)	(2.73)			(8.44)	(2.73)
ROA			−0.028**	0.007			−0.028**	0.007
			(−2.23)	(0.57)			(−2.22)	(0.57)
PPE			−0.095***	−0.073***			−0.095***	−0.074***
			(−29.83)	(−19.58)			(−29.88)	(−19.62)
Growth			−0.008***	−0.005***			−0.008***	−0.005***
			(−5.77)	(−3.35)			(−5.77)	(−3.35)
TQ			0.000	0.000			0.000	0.000
			(0.46)	(0.46)			(0.46)	(0.46)
Age			0.002***	0.001***			0.002***	0.001***
			(21.19)	(15.73)			(21.23)	(15.79)
First			0.000**	0.000***			0.000**	0.000***
			(2.54)	(3.72)			(2.51)	(3.69)
Ins			−0.000***	−0.000***			−0.000***	−0.000***
			(−3.21)	(−3.27)			(−3.18)	(−3.24)
Board			−0.017***	−0.010***			−0.017***	−0.010***
			(−5.53)	(−3.35)			(−5.53)	(−3.35)
Dir			−0.021**	−0.014			−0.021**	−0.014
			(−2.10)	(−1.45)			(−2.11)	(−1.46)
Dual			0.004***	0.002*			0.004***	0.002*
			(3.83)	(1.77)			(3.83)	(1.77)
Constant	0.066***	0.037***	0.080***	0.071***	0.036***	0.013*	0.061***	0.052***
	(16.74)	(4.46)	(6.08)	(4.48)	(70.93)	(1.71)	(4.80)	(3.38)
Industry	NO	YES	NO	YES	NO	YES	NO	YES
Year	NO	YES	NO	YES	NO	YES	NO	YES
*N*	20,647	20,647	20,647	20,647	20,647	20,647	20,647	20,647
Adj-*R*^2^	0.004	0.115	0.085	0.160	0.003	0.115	0.085	0.160

Significance levels are denoted by ***, **, and * for 1, 5, and 10% respectively; *t*-values are presented in parentheses.

Expanding the sample dataset. In the previous section, we screen the dataset by excluding listed companies that were specially treated. However, the excluded data may provide additional insights into our research. Therefore, we re-run the regression using the sample set that includes the specially treated listed companies to ensure the robustness of the results. Table 14 presents the regression results, which indicate that the coefficients of SBD are consistently positive and robust.

**Table 14:** Regression results of expanding the sample dataset.

Variables	(1)	(2)	(3)	(4)
	Fin	Fin	Fin	Fin
SBD	0.013***	0.009***	0.007***	0.006***
	(5.82)	(4.35)	(3.12)	(2.79)
Size			0.001*	−0.000
			(1.66)	(−0.03)
Lev			−0.046***	−0.049***
			(−16.59)	(−16.88)
*CF*			0.058***	0.030***
			(8.23)	(4.33)
ROA			−0.023***	−0.008
			(−3.06)	(−1.01)
PPE			−0.090***	−0.075***
			(−33.95)	(−23.67)
Growth			−0.005***	−0.004***
			(−4.01)	(−3.28)
TQ			0.001***	0.002***
			(3.44)	(3.69)
Age			0.002***	0.002***
			(26.00)	(19.75)
First			0.000***	0.000***
			(4.04)	(4.63)
Ins			−0.000***	−0.000***
			(−5.12)	(−5.51)
Board			−0.014***	−0.010***
			(−5.17)	(−3.62)
Dir			−0.017**	−0.016**
			(−1.98)	(−1.99)
Dual			0.003***	0.002*
			(3.37)	(1.67)
Constant	0.035***	0.012**	0.068***	0.059***
	(78.73)	(2.53)	(6.02)	(4.57)
Industry	NO	YES	NO	YES
Year	NO	YES	NO	YES
*N*	25,640	25,640	25,640	25,640
Adj-*R*^2^	0.002	0.100	0.090	0.152

Significance levels are denoted by ***, **, and * for 1, 5, and 10% respectively; *t*-values are presented in parentheses.

## 5 Further analysis

### 5.1 Digital transformation

The advent of cutting-edge digital technologies, including artificial intelligence, blockchain, big data, and cloud computing, is poised to revolutionize corporate governance through their seamless integration with enterprises. The application of digital technology has enhanced the information environment within enterprises, effectively mitigating the issue of information asymmetry (Niu et al., 2023). By enhancing the transparency of financial information and internal controls (Goldfarb and Tucker, 2019), shareholders are empowered to more effectively monitor operational conditions and inhibit the short-sighted opportunistic behavior of managers (Li et al., 2024). Therefore, as a crucial tool for management and supervision (Obwegeser et al., 2020), digital technology can effectively mitigate the management entrenchment associated with staggered boards, reduce the opportunistic behavior of the management, enhance the efficiency of managerial decision-making processes, and thereby restrain excessive financial investments by enterprises. The application of digital transformation is expected to effectively mitigate the promotional impact of staggered boards on corporate financialization and enhance the efficiency of corporate asset allocation.

According to previous study (Ren et al., 2023; Fang and Ju, 2024), we employ the natural logarithm of the aggregate count of characteristic words related to digital transformation in the Management Discussion and Analysis (MD&A) section of annual reports from listed companies as a metric for quantifying the extent of digital transformation (Digital). Additionally, we utilize a moderation model to examine the governance role played by digital transformation in influencing the impact of staggered boards on corporate financialization. The results are shown in Table 15. It is found that the coefficient of Digital*SBD is significantly negative, indicating that digital transformation can significantly inhibit the promotion effect of staggered boards on corporate financialization.

**Table 15:** Regression results of further analysis.

Variables	Digital	SOEs	Non-SOEs	High Industry Competition	Low Industry Competition
	Fin	Fin	Fin	Fin	Fin
SBD	0.025***	0.003	0.016***	0.015***	0.004
	(3.86)	(0.69)	(4.78)	(5.03)	(0.79)
SBD*Digital	−0.004*				
	(−1.89)				
Digital	−0.005***				
	(−7.71)				
Size	0.001**	0.001*	0.001*	0.000	0.002*
	(2.26)	(1.74)	(1.65)	(0.08)	(1.71)
Lev	−0.060***	−0.074***	−0.060***	−0.061***	−0.049***
	(−17.92)	(−11.48)	(−13.82)	(−16.07)	(−5.70)
*CF*	0.022**	−0.005	0.021**	0.023**	0.029
	(2.54)	(−0.32)	(2.08)	(2.52)	(1.39)
ROA	−0.000	−0.045*	0.023	−0.004	0.040
	(−0.04)	(−1.92)	(1.47)	(−0.30)	(1.25)
PPE	−0.078***	−0.076***	−0.077***	−0.072***	−0.079***
	(−19.12)	(−12.75)	(−15.08)	(−17.71)	(−8.28)
Growth	−0.004***	−0.003	−0.004**	−0.004***	−0.007**
	(−3.02)	(−1.19)	(−2.29)	(−2.71)	(−2.00)
TQ	0.000	0.001	−0.000	0.000	−0.001
	(0.77)	(0.45)	(−0.01)	(0.67)	(−0.96)
Age	0.001***	0.001***	0.002***	0.001***	0.002***
	(16.17)	(7.86)	(10.90)	(13.25)	(9.26)
First	0.000***	0.000	0.000***	0.000**	0.000***
	(3.92)	(1.58)	(4.95)	(2.37)	(3.74)
Ins	−0.000***	−0.000***	−0.000**	−0.000**	−0.000***
	(−3.49)	(−4.59)	(−2.43)	(−2.20)	(−3.28)
Board	−0.010***	−0.020***	−0.002	−0.009***	−0.014*
	(−3.19)	(−4.64)	(−0.41)	(−2.80)	(−1.86)
Dir	−0.016	−0.019	−0.004	−0.009	−0.032
	(−1.53)	(−1.37)	(−0.26)	(−0.84)	(−1.40)
Dual	0.002**	0.006**	0.001	0.002	0.002
	(2.21)	(2.29)	(0.68)	(1.58)	(0.56)
Constant	0.043***	0.075***	0.009	0.059***	0.030
	(2.66)	(3.96)	(0.38)	(3.50)	(0.90)
Industry	YES	YES	YES	YES	YES
Year	YES	YES	YES	YES	YES
*N*	20,281	6,739	13,908	17,325	3,322
Adj-*R*^2^	0.165	0.237	0.164	0.156	0.200

Significance levels are denoted by ***, **, and * for 1, 5, and 10% respectively; *t*-values are presented in parentheses.

### 5.2 Heterogeneity analysis

Firstly, we examine the effect of the different nature of ownership. Enterprises with different ownership structures exhibit significant variations in their business environment and objectives (Chang et al., 2019; Wang et al., 2022; Clò et al., 2023). State-owned enterprises face more pronounced government intervention (Spiller, 1990), necessitating the pursuit of not only economic benefits but also increased social responsibilities during operations (Shleifer and Vishny, 1994; Chu et al., 2024). Due to government regulations, state-owned enterprises are minimally impacted by external market control. In fact, acquisitions of state-owned enterprises without government permission are challenging to execute (Holmen and Nivorozhkin, 2007). Consequently, within state-owned enterprises, the role of staggered boards in resisting threats from the control market is limited. Correspondingly, their ability to consolidate management’s position of control is not readily apparent. Moreover, government regulation also strengthens supervision over management’s business decisions to prevent excessive engagement in financial investment activities that may undermine long-term enterprise value. Therefore, it can be anticipated that the influence of staggered boards on corporate financialization will be more pronounced in non-state-owned enterprises.

We partition the entire sample into state-owned enterprises (SOEs) and non-state-owned enterprises (non-SOEs) and employed model (1) to perform separate regressions for each subset. The results are presented in Table 15. The results show that in the sample of non-state-owned enterprises, the regression coefficient of SBD is significantly positive at 1% level, while it is not significant in the sample of state-owned enterprises which is consistent with the expectation.

Then, we explore the effect of degree of industry competition. Industry competition is widely recognized as a significant external force that influences corporate governance (Kang and Kim, 2021). On the one hand, when faced with intensified industry competition, firms may allocate more resources toward core business investments to enhance their competitive advantage and avoid elimination. This implies that product market competition can effectively mitigate managerial opportunistic behavior (Tang and Chen, 2020) and reduce the extent of corporate financialization. On the other hand, intense industry rivalry tends to diminish profit margins for enterprises’ sold products. Moreover, given that management compensation is linked to profits, it may incentivize managers to invest in financial assets for short-term performance improvement (Bimo et al., 2022), thereby increasing the level of corporate financialization.

According to Fang and Ju (2024), the company’s industry competition level is determined by the median of the HHI (Herfindahl–Hirschman Index) in the same year. Samples below the median are classified as high industry competition sample, while those above are classified as low industry competition sample. We employ model (1) to conduct separate regressions for each cohort (refer to Table 15 for the outcomes). The findings reveal that in enterprises operating within highly competitive industries, the regression coefficient of SBD exhibits a significantly positive association at a significance level of 1%, whereas it lacks statistical significance in enterprises operating within less competitive industries. These results indicate that intense industry competition amplifies management’s impetus to invest in financial assets, thereby accentuating the role of staggered boards in fostering corporate financialization.

## 6 Conclusion and discussion

### 6.1 Conclusion

Based on a sample of A-share listed companies in China from 2011 to 2020, this study empirically examines the impact of staggered boards on corporate financialization. Our results confirm the hypothesis that the establishment of staggered board can isolate the management from market supervision, intensify the entrenchment of management, and divert resources from the activities that can improve the shareholder value, such as the main business investment, therefore improving the level of corporate financialization. The results are consistent with recent research based on the managerial entrenchment view of staggered boards (Faleye, 2007; Karakas and Mohseni, 2021; Tanthanongsakkun et al., 2023; Chatjuthamard et al., 2024). After solving the endogeneity problem by using individual fixed effect model, lagged variable method, propensity score matching (PSM) analysis, Heckman two-stage model and instrumental variable (IV) approach, the results are still robust. The mechanism test reveals that staggered boards intensify the agency conflict between shareholders and managers, increase firms’ agency costs, and subsequently amplify the degree of corporate financialization. On the one hand, staggered board will intensify the speculative psychology and profit motive of board members, causing management to use financial investment to seize private interests. On the other hand, staggered board system will also intensify the laziness psychology of management, potentially motivating management to use financial investment to quickly achieve the target performance without too much effort.

It is necessary to find feasible governance measures to solve the agency problem caused by the staggered board of directors and restrain the growth of the level of corporate financialization. According to agency theory, incentive and supervision are important measures to optimize corporate governance. We find that both managerial ownership and high-quality audit supervision effectively inhibit the promoting effect of staggered boards on corporate financialization.

Our study also provides new insights into the impact of digital transformation on the corporate financialization. We find that digital technology, as an important management and supervision tool (Obwegeser et al., 2020), can effectively alleviate the management entrenchment related to staggered boards of directors, reduce the opportunistic behavior of the management, improve the efficiency of the management decision-making process, and thus inhibit the excessive financial investment of enterprises. In fact, recent studies have demonstrated the inhibitory effect of digital transformation on corporate financialization, and pointed out the improvement of corporate information liquidity and operational capabilities as possible reasons (Zhang et al., 2023; Fang and Ju, 2024). On the basis of further proving this view, our results emphasize that digital transformation can also inhibit the improvement of corporate financialization by influencing/optimizing corporate governance channels.

The impact of staggered boards is likely to vary across firms. Heterogeneity analysis demonstrates that staggered boards play a more prominent role in promoting corporate financialization in non-SOEs and firms facing intense industry competition compared to SOEs and firms with less competition. These findings establish boundary conditions for understanding how the staggered board system affects corporate financialization. Although this study only uses data from China, its conclusions have implications for other countries and regions that are in the stage of governance reform.

### 6.2 Discussion

Our study contributes to several important areas of the current literature.

Firstly, our study contributes to the literature on the factors influencing corporate financialization. Previous studies have shown that corporate financialization is the investment behavior under the joint influence of external environment (Perillo and Battiston, 2020; Zhao and Su, 2022; Yang et al., 2024) and internal governance mechanism (Du et al., 2022; Feng et al., 2022). The staggered board belongs to the category of internal governance mechanism. We also explore how to mitigate the problem of corporate financialization caused by the staggered board system through effective monitoring and incentive mechanisms, with a view to providing insights into the governance of current corporate financialization.

Secondly, this study contributes to the discussion on staggered board systems by examining their impact on the current economic issue of financialization in firms. Although past research has mainly focused on the impact of staggered boards on firm value, recent studies have highlighted the need to examine their impact on other critical firm outcomes, such as corporate investment (Mbanyele, 2021; Chen et al., 2022), carbon emissions (Tanthanongsakkun et al., 2023), and corporate culture (Chatjuthamard et al., 2024). Our study complements previous research on corporate investment perspectives and supports Mbanyele’s (2021) conclusion that staggered boards of directors negatively impact investment quality and efficiency.

Finally, there has always been a fierce dispute regarding the staggered boards. Conventional wisdom holds that the staggered board system can reduce the threat of corporate takeovers (Jiraporn et al., 2012), and enable managers to focus on long-term enterprise development (Cremers et al., 2017), thereby enhancing corporate value (Stráska and Waller, 2016). Nevertheless, some scholars have pointed out that the staggered board may weaken the disciplining effect of external control markets (Bates et al., 2008; Bebchuk et al., 2009), giving rise to the moral hazard of management and management entrenchment (Karakas and Mohseni, 2021) which is detrimental to corporate value creation (Cohen and Wang, 2017). It is demonstrated that increased managerial moral hazard is a significant mechanism by which staggered boards impact corporate financialization. The matter of corporate financialization concerning alterations in board structure aligns with the perspective of managerial entrenchment on antitakeover provisions (Bebchuk and Cohen, 2005; Zhao and Chen, 2008; Mbanyele, 2021; Tanthanongsakkun et al., 2023; Chatjuthamard et al., 2024). Furthermore, similar to Chen et al. (2021), this study demonstrates the existence of a management entrenchment effect of the staggered boards in the Chinese market.

The main implications of this study are as follows.

Firstly, our research has important implications for corporate leaders. On the one hand, companies should pay attention to the significant impact of staggered boards on directors’ investment decision preferences and consider the institutional cost of the staggered boards. While staggered boards excel in maintaining corporate governance stability and management continuity, they also mitigate the adverse effects of failed financial investments on executives’ careers, potentially at the expense of exacerbating irrational investments and depleting company value. Considering the heterogeneity of the impact of staggered board on different enterprises, companies should carefully consider whether to introduce staggered board on the basis of a reasonable assessment of their own corporate governance, so as to avoid blindly following the trend and damaging the sustainable and healthy development of enterprises. On the other hand, the defense mechanism established by staggered board systems serves as a “relative” rather than an “absolute” defense. Companies should establish robust and effective internal governance mechanisms and enhance existing supervision and incentive systems to fundamentally curb unjustifiable management investment decisions and promote high-quality business development. In addition, companies should moderately promote the digital transformation. With the integration of digital technology and corporate operations, the efficiency of corporate governance can be effectively enhanced.

Secondly, our study provides important insights for policymakers. On the one hand, policymakers should provide reasonable guidance on the anti-takeover behavior of listed companies, amend relevant provisions of the law on anti-takeover clauses, make up for shortcomings in anti-takeover legislation, and promote the sustainable and healthy development of the capital market. On the other hand, the government should actively guide the return of capital to its source, formulate policies and measures that contribute to the sustainable development of real enterprises, guide real enterprises to make real investment by strengthening credit activities, and enhance the capability of financial services for the real economy.

Finally, our study has important implications for regulators. On the one hand, regulators should further improve the identification and supervision mechanism of the management defense clauses formulated by enterprises, especially strengthen the supervision and examination of enterprises with weak internal and external governance environment, and timely identify and stop the behavior that harms investors’ interests. On the other hand, regulators should actively implement the supervision of the financial investment of non-financial corporates, strengthen the financial supervision and examination after the information disclosure, and prevent the excessive flow of funds into the virtual economy.

However, there are several limitations to be acknowledged in this study. Firstly, our study was conducted primarily in the Chinese market. Although it has important implications for emerging markets and regions with rapidly developing financial markets, expanding the study to different geographic regions and markets could improve the generalizability of the findings. Secondly, although the mechanism analysis primarily focuses on agency conflicts, it is important to consider other potential mechanisms that might have been overlooked. Thirdly, we primarily employ an empirical modelling approach to examine the correlation between staggered boards and corporate financialization. In the future, we could expand our research through methods such as qualitative research or case studies. Furthermore, the implementation of qualitative indicators to evaluate agency conflicts could enhance the comprehension of the mechanisms through which staggered boards impact the financialization of companies. Lastly, while heterogeneity analysis has been conducted considering ownership structure and industry competition, future studies could further explore different governance models and macroeconomic environment perspectives.

## Data availability statement

The raw data supporting the conclusions of this article will be made available by the authors, without undue reservation.

## Author contributions

CC: Conceptualization, Methodology, Resources, Supervision, Writing – review & editing, Writing – original draft. YZ: Conceptualization, Data curation, Formal analysis, Methodology, Software, Visualization, Writing – original draft, Writing – review & editing.

## Footnotes

^1^These conclusions are generated based on the analysis of 20,647 samples included in this study.
